# Impact of *KMT2A* Rearrangement on Peripheral T-Cell Lymphoma, Not Otherwise Specified, and Angioimmunoblastic T-Cell Lymphoma

**DOI:** 10.3390/biomedicines13102347

**Published:** 2025-09-25

**Authors:** Tong-Yoon Kim, Tae-Jung Kim, Eun Ji Han, Gi-June Min, Seok-Goo Cho, Youngwoo Jeon

**Affiliations:** 1 Department of Hematology, Yeouido St. Mary’s Hospital, College of Medicine, The Catholic University of Korea, Seoul 07345, Republic of Korea; tyk@catholic.ac.kr; 2 Lymphoma and Cell Therapy Research Center, Yeouido St. Mary’s Hospital, College of Medicine, The Catholic University of Korea, Seoul 07345, Republic of Korea; 3 Department of Hospital Pathology, Yeouido St. Mary’s Hospital, College of Medicine, The Catholic University of Korea, Seoul 07345, Republic of Korea; 4 Division of Nuclear Medicine, Department of Radiology, Yeouido St. Mary’s Hospital, College of Medicine, The Catholic University of Korea, Seoul 07345, Republic of Korea; 5 Department of Hematology, Seoul St. Mary’s Hospital, College of Medicine, The Catholic University of Korea, Seoul 06591, Republic of Korea

**Keywords:** Angioimmunoblastic T-cell lymphoma, peripheral T-cell lymphoma, non-Hodgkin lymphomas, KMT2A rearrangement

## Abstract

**Background**: Angioimmunoblastic T-cell lymphoma (AITL) and peripheral T-cell lymphomas (PTCL), not otherwise specified (NOS), share overlapping histology and T-follicular helper (TFH) biology but often show divergent outcomes and treatment needs. The clinical significance of *KMT2A* rearrangement (*KMT2A*-r) in nodal PTCL remains undefined. We aimed to investigate the clinicogenomic features and prognostic impact of *KMT2A*-r in AITL and PTCL-NOS. **Methods**: We retrospectively analyzed consecutive patients diagnosed with AITL or PTCL-NOS between 2021 and 2024 at two centers. All patients underwent 523-gene DNA/RNA next-generation sequencing. Gene co-variation and diagnostic splits were summarized using network and decision-tree analyses. **Results**: Overall, 37 patients were included (AITL: 14; PTCL-NOS: 23), with similar baseline clinical characteristics. In AITL, TFH markers were more frequently expressed, and *RHOA* mutations were enriched. *KMT2A*-r occurred in 24% of cases without histology-specific enrichment. AITL showed better 2-year overall survival (OS) than PTCL-NOS (70.7% vs. 38.8%; *p* = 0.040) but similar progression-free survival (PFS). Univariate analysis revealed that *KMT2A*-r, lactate dehydrogenase elevation, and bone-marrow involvement predicted inferior PFS (Hazard ratio for *KMT2A*-r: 2.56). Median PFS was 5.9 versus 12.5 months in the *KMT2A*-r and non-*KMT2A*-r groups, respectively (*p* = 0.039). Brentuximab vedotin (BV) plus cyclophosphamide, doxorubicin, and prednisone did not significantly improve OS or PFS overall; however, exploratory analysis indicated improved PFS in the *KMT2A*-r subset. **Conclusions**: *KMT2A*-r delineates an adverse-risk biology in nodal PTCL, aligns with non-TFH genomic hubs and markers of tumor burden, and may serve as a stratifier and hypothesis-generating target for BV-based strategies.

## 1. Introduction

Peripheral T-cell lymphomas (PTCL) account for 5–20% of non-Hodgkin lymphomas and are relatively more frequent in Asia. In Asia, PTCL, not otherwise specified (PTCL-NOS) and angioimmunoblastic T-cell lymphoma (AITL) represent the second and third most common PTCL subtypes [1]. Histologically, they partly overlap, and diagnostic reproducibility is imperfect even among experts [2]. The 2016 WHO revision and subsequent 5th edition emphasize the concept of a T-follicular helper (TFH) lineage for AITL and a subset of PTCL-NOS, defined by variable expression of PD-1, ICOS, CXCR5, CD10, and/or BCL6. These lymphomas are usually accompanied by a distinctive microenvironment with arborizing high-endothelial venules, follicular dendritic cell meshwork, and EBV-positive B-immunoblasts [3,4]. The TFH framework is supported genomically by frequent mutations in *TET2* and *DNMT3A*, the *IDH2* R172 hotspot, and the Ras homolog family member A (*RHOA*) G17V switch, often co-occurring and tightly linked to TFH differentiation [5,6,7,8]. Patients with TFH lymphomas have shown sustained response to hypomethylating agents and proximal TCR pathway inhibition, whereas non-TFH PTCL-NOS exhibit distinct dependencies [9,10]. These genetic differences in tumors may impact chemotherapy sensitivity.

This study aimed to explore the contribution of *KMT2A* rearrangements (*KMT2A*-r) to nodal PTCL biology, which remains poorly defined. Although *KMT2A*-r is a founding lesion in acute leukemias, mechanistic and clinical data in mature T-cell neoplasms are scarce [11]. Using next-generation sequencing (NGS), we conducted a bi-center analysis to identify clinicopathologic and genomic features across AITL and PTCL-NOS and assess the prognostic impact of *KMT2A*-r and treatment options in first-line chemotherapy.

## 2. Materials and Methods

### 2.1. Patient Selection

We conducted a retrospective cohort study of patients diagnosed with de novo AITL or PTCL-NOS between January 2021 and September 2024 at Seoul St. Mary’s Hospital and Yeouido St. Mary’s Hospital. All biopsies were reviewed by hematopathologists with expertise in T-cell lymphomas. This study was approved by the Institutional Review Board and Ethics Committee of the Catholic Medical Center, Republic of Korea (XC25RIDI0050). The requirement for informed consent was waived because only anonymized archival material and routine clinical data were used.

### 2.2. NGS

Targeted NGS was conducted using the QIAseq Pan-cancer Multimodal Panel (Qiagen, Hilden, Germany), which assesses DNA aberrations spanning 523 cancer-associated genes. At diagnosis, RNA and DNA were extracted from Formalin-fixed, paraffin-embedded (FFPE) tissue blocks using the QIAamp DNA FFPE Tissue Kit (Qiagen, Hilden, Germany) according to the manufacturer’s protocol. Input DNA quantity and integrity were evaluated using the QIAseq DNA QuantiMIZE Array (Qiagen, Hilden, Germany), ensuring that the material met the requirements for downstream library construction.

Library construction was performed according to the QIAseq Multimodal Panel HT Handbook (Qiagen). The workflow comprised enzymatic DNA fragmentation, end-repair, A-tailing (adenine overhang addition), adapter ligation, and polymerase chain reaction (PCR) amplification. Unique molecular identifiers were incorporated during library building to minimize PCR duplicates and sequencing artifacts, thereby improving the accuracy of variant detection. Libraries were sample-indexed, quantified using quantitative PCR, and quality-checked on an Agilent Bioanalyzer (Agilent Technologies, Santa Clara, CA, USA) to confirm fragment-size distribution and effective adapter trimming.

Sequencing was performed on Illumina instruments (Illumina, San Diego, CA, USA) using Qiagen-supplied custom sequencing primers. Primary data processing and variant calling were executed in the QIAGEN CLC Genomics Workbench v25.0 (QIAGEN Aarhus A/S, Aarhus, Denmark), and downstream variant annotation and clinical interpretation were performed using QIAGEN Clinical Insight Interpret v9.3.2. A comprehensive list of all molecular alterations detected in the cohort is provided in Supplementary Table S1.

### 2.3. Therapy and Response Assessment

First-line therapy was selected at the physician’s discretion and included CHOP/CHOEP, ProMACE-CytaBOM, or brentuximab vedotin (BV) plus cyclophosphamide, doxorubicin, and prednisone (CHP) [12,13,14]. Regimen selection was performed based on age, comorbidity, CD30 expression, and performance status. BV-CHP was offered to CD30-positive cases and, in select circumstances, to patients with borderline/equivocal CD30 expression or when CD30 results were pending at treatment initiation.

Responses were evaluated according to the Lugano 2014 criteria. Consolidation with autologous stem-cell transplantation (ASCT) was considered for fit patients attaining complete remission (CR). Staging was consistently evaluated using the Lugano classification and Deauville score. CR was defined as the disappearance of target lesions on computed tomography with normalization of 18F-fluorodeoxyglucose positron emission tomography-computed tomography uptake at all sites (Deauville score, 1–3) [15]. BuMelTT protocols (busulfan, melphalan, and thiotepa) were initiated as previously described [16].

### 2.4. Statistical Analysis

Survival endpoints were overall survival (OS) and progression-free survival (PFS). OS was measured from diagnosis to death from any cause; patients alive at last contact were censored at that date. The primary time-to-event endpoint was relapse-based PFS, defined as the time from diagnosis to relapse/progression; deaths without documented progression were censored at the date of death. Initiation of autologous stem-cell transplantation (ASCT) used as frontline consolidation was not considered an event. Patients therefore remained at risk for relapse after ASCT, and relapses occurring post-ASCT were counted as events. In a sensitivity analysis, we re-estimated curves censoring at the time of ASCT; the conclusions were unchanged. Curves were generated using Kaplan–Meier estimates with log-rank tests. Univariate Cox models were used to screen clinical and genomic variables. We defined the multiplicity ‘family’ as the set of 20 univariate tests per endpoint (PFS or OS). False discovery rate was controlled using the Benjamini–Hochberg procedure; BH-adjusted *q*-values are reported alongside raw *p*-values. Given the small cohort, all univariate analyses were pre-specified as exploratory; interpretation emphasized effect sizes and 95% confidence intervals rather than dichotomous *p*-value thresholds. For the multivariable Cox models, covariates that met the univariate screening criterion (*p* < 0.05) were entered to assess their independent associations with OS and PFS. Pearson correlation matrices were constructed to evaluate variable co-movement and visualized using heatmaps. Network analysis was performed using the igraph package (version 2.1.4), with absolute correlation as edge weights; node degree and betweenness were used to identify hubs [17]. A decision tree (package named rpart, version 4.1.24) based on binary features (1 = present, 0 = absent) was used to distinguish AITL from PTCL-NOS. To reveal multi-gene rules in this small cohort, we report the maximal, unpruned tree (very low cp; reduced minsplit/minbucket; increased maxdepth), showing node counts and class probabilities [18]. All analyses were performed using R version 4.2.3 software (R Core Team, Vienna, Austria), and two-sided *p*-values < 0.05 were considered statistically significant.

## 3. Results

### 3.1. Patient Cohort, Baseline Features, and Survival Outcomes

A total of 37 patients were included in the study (AITL n = 14; PTCL-NOS n = 23). The median age was 61 years (interquartile range: 54–69), and 18 (48.6%) patients were women. Most patients had advanced-stage disease (stage III–IV, 91.9%), and 40.5% had bone-marrow involvement. LDH levels were elevated above the upper limit of normal in 62.2% of cases. TFH markers, including CD10, BCL6, and PD-1, were significantly more frequent in AITL than in PTCL-NOS. Additionally, *RHOA* mutations were enriched in AITL (95.7% vs. 57.1%; *p* = 0.014). *KMT2A*-r was present in nine patients (9/37, 24.3%), without strong enrichment based on histology. Additional baseline characteristics are presented in Table 1.

**Figure 1:**
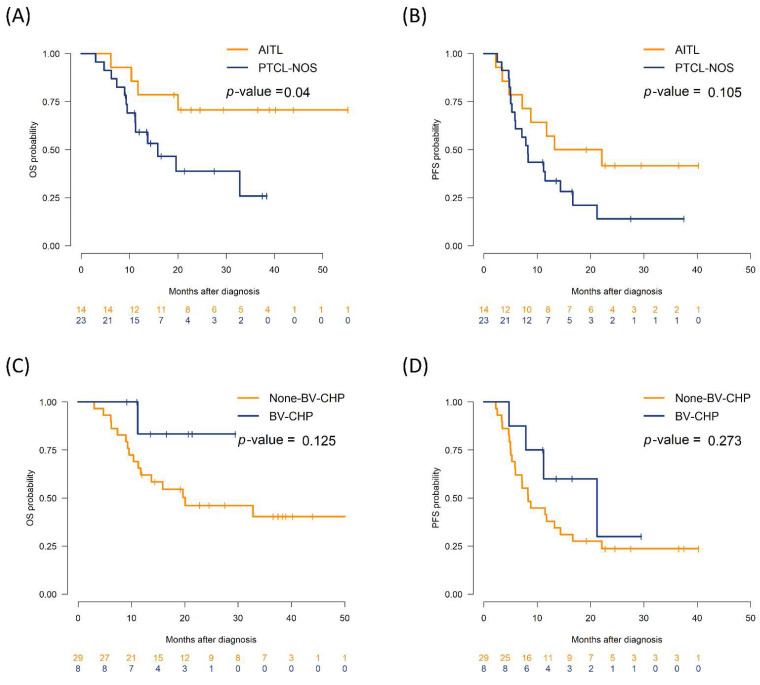
Survival according to histology and initial regimen. Kaplan–Meier curves for OS and PFS by (**A**,**B**) histology (AITL vs. PTCL-NOS), (**C**,**D**) initial regimen (BV-CHP vs. non-BV-CHP) Tick marks denote censoring; numbers at risk are shown below the axes.

At a median follow-up of 24 months among survivors, 2-year OS was higher in AITL versus PTCL-NOS (70.7% vs. 38.8%; *p* = 0.040), whereas PFS was similar. BV-CHP did not significantly improve OS or PFS in the overall population, and differences in CD30 did not affect treatment outcomes (Figure 1). This is consistent with uncertainty regarding the benefit of BV-CHP in PTCL subtypes except for anaplastic large cell lymphoma [19]. Patients undergoing ASCT consolidation had longer OS (*p* = 0.01) and PFS (*p* = 0.039) than those managed without ASCT. Although improved survival outcomes with ASCT have been consistently reported, careful interpretation is required owing to the risk of selection bias favoring chemotherapy responders [20,21].

### 3.2. Impact of Genetic Alteration on Survival Outcomes

**Figure 2:**
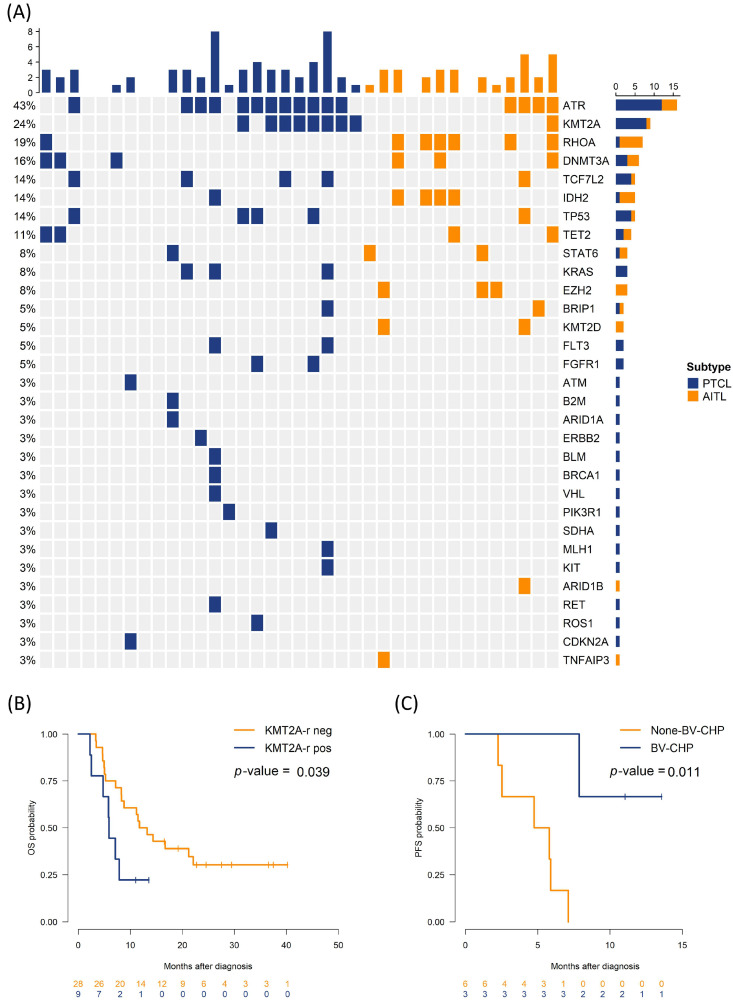
Genomic landscape and outcome by *KMT2A* rearrangement (*KMT2A*-r). (**A**) Oncoprint summarizing alteration frequencies (rows) across cases (columns), colored based on histology. (**B**) OS by *KMT2A*-r (positive vs. negative). (**C**) PFS by regimen (BV-CHP vs. non-BV-CHP) in *KMT2A*-r positive group. neg, negative; pos, positive.

Across the cohort, *ATR* mutations were the most frequent (43.2%), followed by *KMT2A*-r, *RHOA*, *DNMT3A*, and *IDH2* (Figure 2A). Univariate Cox analyses revealed that LDH elevation (hazard ratio [HR]: 3.10, 95% confidence interval [CI]: 1.27–7.58), bone marrow involvement (HR: 2.98, 95% CI: 1.25–7.08), and *KMT2A*-r (HR: 2.56, 95% CI: 1.02–6.45) predicted shorter PFS. *DNMT3A* mutation, ECOG ≥ 2, LDH elevation trended toward inferior OS (Table 2 and Table S2). *DNMT3A* mutations often correlated with higher ECOG and older age, both of which contribute to poorer OS. This finding aligns with prior reports that clinical indices (e.g., ECOG, LDH) affect survival outcomes across PTCL subtypes [4]. Median PFS was 5.9 months in patients with *KMT2A*-r, compared to 12.5 months in those without *KMT2A*-r (log-rank *p* = 0.039). Figure 2B,C illustrate survival according to *KMT2A*-r and treatment regimen. Among the six patients with *KMT2A*-r who received BV-CHP, median PFS was not reached at 12 months, compared with 4.8 months (95% CI: 2.1–7.5) in three patients with *KMT2A*-r treated with other regimens (*p* = 0.011); however, numbers are small, with wide confidence intervals. Baseline CD30 levels did not differ substantially between the *KMT2A*-r and non-*KMT2A*-r groups. In multivariable models, PFS estimates remained adverse but non-significant—KMT2A-r (HR = 1.65, 95% CI 0.59–4.59, *p* = 0.34), LDH (HR = 2.32, 95% CI 0.78–6.94, *p* = 0.131), bone-marrow involvement (HR = 1.45, 95% CI 0.46–4.55, *p* = 0.522). For OS, ECOG ≥ 2 remained significant (HR = 5.24, 95% CI 1.28–21.5, *p* = 0.021) and DNMT3A trended adverse (HR = 2.62, 95% CI 0.93–7.36, *p* = 0.069, Table S3).

### 3.3. Interrelationship Among the Molecular Landscape

**Figure 3:**
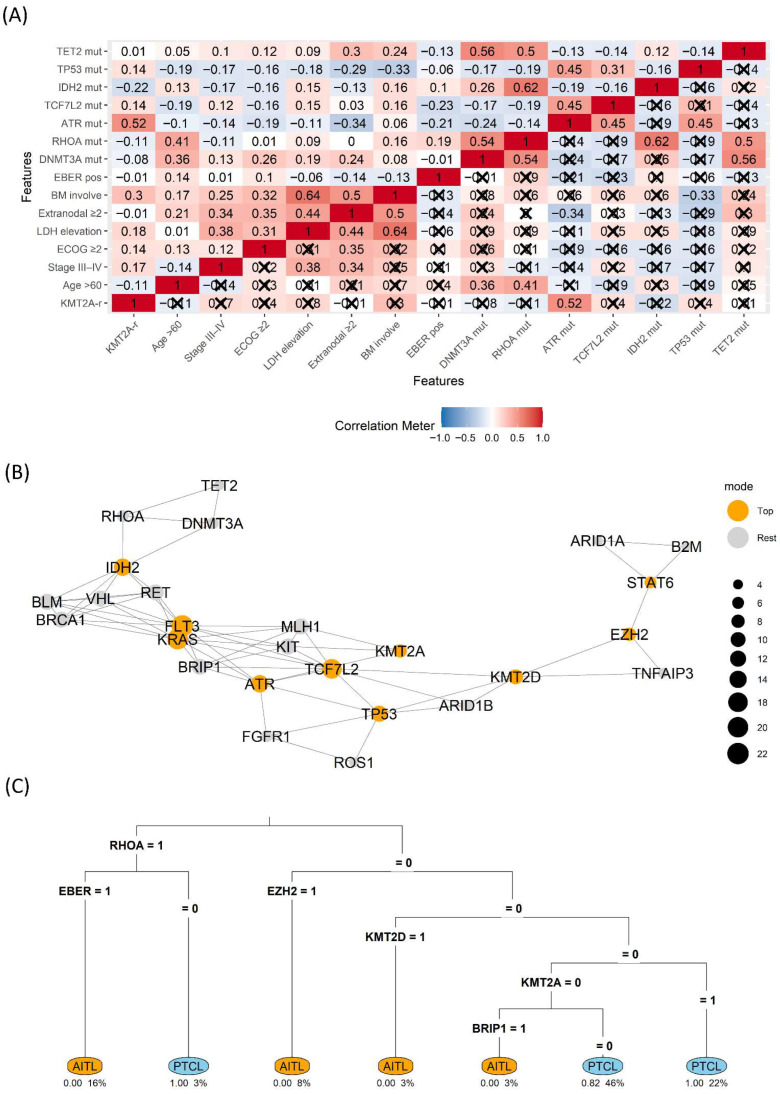
Clinicogenomic map (**A**) Correlation heatmap across clinical and genomic features. Tiles encode Pearson’s r (color scale). Cells not reaching two-sided *p* < 0.05 are marked with a cross (×); symbols are displayed on the lower triangle only to avoid duplication. (**B**) Gene co-variation network with betweenness highlighting (Top vs. Rest) (**C**) rpart decision tree separating AITL vs. PTCL using binary features (1 = present).

Heatmap analysis revealed a cluster linking *KMT2A*-r with ATR mutation (correlation coefficient = 0.52), bone marrow involvement (correlation coefficient = 0.3) and, elevated LDH (correlation coefficient = 0.14), whereas the canonical TFH cluster (*RHOA*, *IDH2*, *TET2/DNMT3A*) tended to anti-correlate with *KMT2A*-r. In the network view, *KMT2A* and *ATR* emerged as high-betweenness nodes bridging a DNA-damage repair motif (*ATR–MLH1*) and receptor tyrosine-kinase signaling (*KIT*), suggestive of crossed stress and growth cues. The decision tree split first on *RHOA* (1 → *AITL*), then used EBV-encoded RNA in situ hybridization and *EZH2* for refinement. Within the *RHOA* = 0/*EZH2* = 0 branch, *KMT2A* = 1 predicted PTCL classification, indicating that *KMT2A* is informative, particularly when TFH signals are absent (Figure 3).

## 4. Discussion

This two-center study integrated clinicopathologic and genomic data in patients with PTCL-NOS or AITL to examine the impact of *KMT2A*-r in nodal PTCL. Our findings align with established TFH biology in AITL and its overlap with PTCL-NOS. Sequencing studies have shown that *RHOA* mutations frequently co-occur with mutations in *TET2* (and often *DNMT3A*), and that *IDH2* mutations provide a convergent methylation program; together these lesions drive TFH differentiation and the characteristic microenvironment [22]. The Human Pathology multicenter study further demonstrated that increasing numbers of positive TFH markers track with AITL-like histology and that PTCL-TFH overlaps with, but is not identical to, AITL [23]. Within that framework, our data place *KMT2A*-r outside the TFH cluster and closer to non-TFH PTCL-NOS behavior—consistent with shorter PFS and higher tumor-burden markers.

*KMT2A* rearrangements have been sparsely investigated in PTCLs. In experimental models, enforced expression of the KMT2A–AF9 fusion is sufficient to induce leukemia, highlighting the strong oncogenic potential of KMT2A fusion proteins [24]. In our cohort, *KMT2A*-rearranged cases correlated with elevated LDH and bone-marrow involvement, features that typically accompany advanced disease. Moreover, correlation-network analysis revealed that *KMT2A* alterations frequently co-occurred with other genetic events (for example, ATR), consistent with a heavier overall genomic alteration burden.

Although ASCT in first remission was associated with superior survival in our cohort, a major practical challenge is that durable CR is not reliably achieved with current induction regimens. Therefore, prolonging the first PFS—by selecting front-line chemotherapy that delivers rapid, deep, and sustained disease control—becomes the key determinant of long-term outcome.

BV is an antibody–drug conjugate that targets CD30 and delivers the microtubule poison Monomethyl auristatin E after receptor-mediated internalization, culminating in mitotic arrest and apoptosis. In ECHELON-2, adding BV to CHP significantly improved OS in CD30-positive PTCL; however, the trial was dominated by Anaplastic large cell lymphoma (ALCL), leaving the effect size in non-ALCL subtypes (AITL and PTCL-NOS) less certain. Moreover, across non-ALCL cohorts the predictive value of CD30 intensity has been inconsistent, and CD30 negativity does not invariably preclude benefit [13,25]. In our cohort, survival outcomes did not differ by CD30 status, reinforcing the view that CD30 expression—while biologically relevant to the mechanism of BV—may be an imperfect surrogate for clinical benefit in AITL or PTCL-NOS. The positive signal for BV-CHP in *KMT2A*-r cases—although exploratory and sample-limited—supports the hypothesis that microtubule-disrupting payloads may be particularly effective when the DNA damage response (DDR) stress is high [26].

Regarding future treatment strategies, menin inhibitors such as revumenib, which is currently used for the treatment of *KMT2A*-rearranged leukemias, may be a potential option [27]. From a biology-driven standpoint, 5-azacitidine—alone or combined with romidepsin—has shown activity in TFH-derived nodal PTCL (AITL and PTCL-NOS with a TFH phenotype), in which epigenetic lesions involving *DNMT3A* and *TET2* are frequent [9,28,29]. Given its comparatively low-intensity profile, this epigenetic approach is a rational option for older, poor-performance patients with *DNMT3A*-mutated PTCL-NOS. In contrast, in our series, *KMT2A*-rearranged cases clustered away from the canonical TFH program, suggesting limited sensitivity to hypomethylating or histone deacetylase inhibitor combinations and a need for alternative strategies.

This study has certain limitations, including the small sample size, retrospective design, heterogeneous frontline regimens, and the lack of centralized pathologic review for all cases. Additionally, NGS was uniform but targeted; thus, structural variants outside the panel’s scope may have been under-captured. Moreover, our exploratory network and decision-tree analyses illustrate associations rather than causation. Nonetheless, the integration of molecular profiling, immunohistochemistry, and outcomes provides a coherent hypothesis: *KMT2A*-r identifies a clinically aggressive, TFH-sparse subset of nodal PTCL in which BV-CHP and ASCT may attenuate risk. On the basis of our data and the rarity of *KMT2A*-r in nodal PTCL, we do not advocate a universal, stand-alone *KMT2A* assay for all patients. Instead, when targeted DNA/RNA next-generation sequencing is employed, *KMT2A* could be included in the panel and prioritized in non-AITL PTCL-NOS or TFH-negative/ambiguous phenotypes and in patients with high-risk clinical features. In canonical AITL/TFH-positive disease, reflex single-gene *KMT2A* testing is optional and may reasonably be deferred to research or clinical-trial settings. Under current practice, our findings support risk stratification and trial referral rather than changes to frontline diagnostic algorithms.

Prospective, multicenter validation is needed to refine the prognostic impact of *KMT2A*-r, delineate partner genes and breakpoint biology, and evaluate tailored regimens, while recognizing that lineage-specific context will determine translatability.

## 5. Conclusions

Our data position *KMT2A*-rearranged nodal PTCL largely outside the canonical TFH program and indicate co-variation with DDR and RTK modules in descriptive network analyses. Given the small cohort, these findings are hypothesis-generating rather than causal. Clinically, the association with elevated LDH, bone-marrow involvement, and shorter PFS argues for selective inclusion of KMT2A within multigene NGS panels to aid risk stratification and trial referral, pending validation in larger series.

## Figures and Tables

**Table 1:** Baseline characteristics according to histology (AITL vs. PTCL-NOS).

Variables	Total (n = 37)	AITL (n = 14)	PTCL (n = 23)	*p*-Value
Sex, n (%)				0.64
Female	18 (48.6)	8 (57.1)	10 (43.5)	
Male	19 (51.4)	6 (42.9)	13 (56.5)	
Diagnosed Age				0.76
≤60 years	21 (56.8)	7 (50.0)	14 (60.9)	
>60 years	16 (43.2)	7 (50.0)	9 (39.1)	
Ann Arbor stage				0.65
I–II	3 (8.1)	2 (14.3)	1 (4.3)	
III–IV	34 (91.9)	12 (85.7)	22 (95.7)	
ECOG2				0.698
<2	32 (86.5)	13 (92.9)	19 (82.6)	
≥2	5 (13.5)	1 (7.1)	4 (17.4)	
Lactate dehydrogenase				0.124
Normal	14 (37.8)	8 (57.1)	6 (26.1)	
Elevated	23 (62.2)	6 (42.9)	17 (73.9)	
Extranodal site involvement				0.76
<2	16 (43.2)	7 (50.0)	9 (39.1)	
≥2	21 (56.8)	7 (50.0)	14 (60.9)	
Bone marrow involvement				0.417
Negative	22 (59.5)	10 (71.4)	12 (52.2)	
Positive	15 (40.5)	4 (28.6)	11 (47.8)	
The International Prognostic Index for Non-Hodgkin’s lymphoma				0.526
Low or Low-Intermediate risk	20 (54.1)	9 (64.3)	11 (47.8)	
High-Intermediate or High risk	17 (45.9)	5 (35.7)	12 (52.2)	
T-follicular helper type				0.168
Negative	32 (86.5)	14 (100.0)	18 (78.3)	
Positive	5 (13.5)	0 (0.0)	5 (21.7)	
Frontline regimen				0.699
BV-CHP	8 (21.6)	2 (14.3)	6 (26.1)	
CHOP/CHOEP	17 (45.9)	7 (50.0)	10 (43.5)	
ProMACE-CytaBOM	12 (32.4)	5 (35.7)	7 (30.4)	
ASCT				0.083
No	26 (70.3%)	7 (50.0%)	19 (82.6%)	
Yes	11 (29.7%)	7 (50.0%)	4 (17.4%)	
**Immunophenotype**				
Epstein–Barr virus-encoded RNAs				0.14
Negative	12 (32.4)	2 (14.3)	10 (43.5)	
Positive	25 (67.6)	12 (85.7)	13 (56.5)	
CD30				0.394
Negative	29 (80.6)	9 (69.2)	20 (87.0)	
Positive	7 (19.4)	4 (30.8)	3 (13.0)	
CD10				0.001
Negative	26 (74.3)	5 (38.5)	21 (95.5)	
Positive	9 (25.7)	8 (61.5)	1 (4.5)	
CD21				0.001
Negative	19 (54.3)	1 (7.1)	18 (85.7)	
Positive	16 (45.7)	13 (92.9)	3 (14.3)	
CD23				0.011
Negative	10 (45.5)	3 (21.4)	7 (87.5)	
Positive	12 (54.5)	11 (78.6)	1 (12.5)	
BCL6				0.004
Negative	13 (39.4)	1 (7.1)	12 (63.2)	
Positive	20 (60.6)	13 (92.9)	7 (36.8)	
PD1				0.014
Negative	9 (25.7)	0 (0.0)	9 (42.9)	
Positive	26 (74.3)	14 (100.0)	12 (57.1)	
Ki-67 proliferation index	64.9 ± 21.8	65.4 ± 22.9	64.6 ± 21.7	0.917
**Next-generation sequencing**				
*ATR*				0.288
Unmutated	21 (56.8)	10 (71.4)	11 (47.8)	
Mutated	16 (43.2)	4 (28.6)	12 (52.2)	
*KMT2A* rearrangement				0.132
Negative	28 (75.7)	13 (92.9)	15 (65.2)	
Positive	9 (24.3)	1 (7.1)	8 (34.8)	
*RHOA*				0.014
Unmutated	30 (81.1)	8 (57.1)	22 (95.7)	
Mutated	7 (18.9)	6 (42.9)	1 (4.3)	
*DNMT3A*				0.833
Unmutated	31 (83.8)	11 (78.6)	20 (87.0)	
Mutated	6 (16.2)	3 (21.4)	3 (13.0)	
*TCF7L2*				0.698
Unmutated	32 (86.5)	13 (92.9)	19 (82.6)	
Mutated	5 (13.5)	1 (7.1)	4 (17.4)	
*IDH2*				0.111
Unmutated	32 (86.5)	10 (71.4)	22 (95.7)	
Mutated	5 (13.5)	4 (28.6)	1 (4.3)	
*TP53*				0.698
Unmutated	32 (86.5)	13 (92.9)	19 (82.6)	
Mutated	5 (13.5)	1 (7.1)	4 (17.4)	
*TET2*				>0.999
Unmutated	33 (89.2)	12 (85.7)	21 (91.3)	
Mutated	4 (10.8)	2 (14.3)	2 (8.7)	

ECOG, Eastern Cooperative Oncology Group; BV-CHP, brentuximab vedotin + cyclophosphamide, doxorubicin, prednisone; ASCT, autologous stem-cell transplantation; AITL, Angioimmunoblastic T-cell lymphoma; PTCL-NOS, peripheral T-cell lymphomas, not otherwise specified; *RHOA*, the Ras homolog family member A. For immunohistochemical markers (CD10, CD21, CD23, BCL6, PD-1), denominators differ from the total cohort owing to missing assessments (percentages are based on the number evaluated). Binary variables in the working dataset were coded as 0 = No and −1 = Yes; they are presented here as “Yes/No”. Ki-67 values are mean ± standard deviation. *p* values are from χ^2^ or Fisher’s exact tests for categorical variables and t-tests for continuous variables. Percentages were computed within each histologic stratum; *p* values were obtained from χ^2^ or Fisher’s exact tests, as appropriate.

**Table 2:** Univariate Cox analysis for progression-free survival.

Variables	HR (95% CI)	*p*-Value	BH-FDR *q*-Value
PTCL-NOS vs. AITL	2 (0.85, 4.68)	0.111	0.317
Female vs. Male	0.52 (0.23, 1.14)	0.102	0.317
Age > 60 years vs. ≤60	1.01 (0.47, 2.19)	0.979	0.979
Ann Arbor stage, III–IV vs. I–II	3.14 (0.42, 23.4)	0.263	0.386
ECOG PS ≥ 2 vs. <2	2.62 (0.86, 8.03)	0.092	0.317
LDH elevation vs. normal	3.1 (1.27, 7.58)	0.013	0.136
Extranodal site involvement, ≥2 vs. <2	1.57 (0.71, 3.49)	0.266	0.386
Bone marrow involvement, positive vs. negative	2.98 (1.25, 7.08)	0.014	0.136
IPI score ≥3 vs. <3	1.74 (0.79, 3.82)	0.167	0.339
TFH phenotype, yes or no	1.74 (0.65, 4.68)	0.269	0.386
Frontline regimen: BV-CHP vs. others	0.56 (0.19, 1.61)	0.28	0.386
EBER positive vs. negative	0.56 (0.25, 1.28)	0.169	0.339
*ATR* mutation vs. unmutated	1.48 (0.68, 3.22)	0.327	0.409
*KMT2A* rearranged, yes vs. no	2.56 (1.02, 6.45)	0.046	0.306
*RHOA* mutation vs. unmutated	1.33 (0.53, 3.33)	0.542	0.603
*DNMT3A* mutation vs. unmutated	2.28 (0.90, 5.81)	0.083	0.317
*TCF7L2* mutation vs. unmutated	1.23 (0.42, 3.61)	0.705	0.742
*IDH2* mutation vs. unmutated	2.05 (0.75, 5.56)	0.16	0.339
*TP53* mutation vs. unmutated	1.58 (0.54, 4.65)	0.402	0.473
*TET2* mutation vs. unmutated	1.79 (0.61, 5.30)	0.29	0.386

AITL, angioimmunoblastic T-cell lymphoma; PTCL-NOS, peripheral T-cell lymphoma, not otherwise specified; BV-CHP, brentuximab vedotin + cyclophosphamide, doxorubicin, and prednisone; LDH, lactate dehydrogenase; ECOG PS, Eastern Cooperative Oncology Group performance status; IPI, International Prognostic Index; TFH, T-follicular helper; EBER, EBV-encoded RNA in situ hybridization; *RHOA*, the Ras homolog family member A; HR, hazard ratio; CI, confidence interval; BH-FDR *q*-value, an adjusted *p*-value calculated using the Benjamini–Hochberg method to control the False Discovery Rate.

## Data Availability

The original data presented in the study are openly available in Dryad (Dataset DOI: 10.5061/dryad.c866t1gm9).

## Supplementary Materials

The following supporting information can be downloaded at: https://www.mdpi.com/article/10.3390/biomedicines13102347/s1, Table S1: Molecular alteration in the study cohort; Table S2: Univariate analysis of the overall survival outcomes of patients with PTCL-NOS and AITL; Table S3: Multivariate analysis of the survival outcomes of patients with PTCL-NOS and AITL.

## Abbreviations

The following abbreviations are used in this manuscript:

AITL	Angioimmunoblastic T-cell lymphoma
PTCL-NOS	Peripheral T-cell lymphoma, not otherwise specified
*RHOA*	Ras homolog family member A
NGS	Next-generation sequencing
FFPE	Formalin-fixed, paraffin-embedded
PCR	Polymerase chain reaction
DDR	DNA damage response
BV	Brentuximab vedotin
BV-CHP	Brentuximab vedotin plus cyclophosphamide, doxorubicin, and prednisone
ASCT	Autologous stem cell transplantation
TFH	T-follicular helper (cell)
LDH	Lactate dehydrogenase
ECOG PS	Eastern Cooperative Oncology Group performance status
ALCL	Anaplastic large cell lymphoma
EBER	EBV-encoded RNA in situ hybridization
IPI	International Prognostic Index
HR	Hazard ratio
CI	Confidence interval
PFS	Progression-free survival
OS	Overall survival
*KMT2A*-r	*KMT2A* rearrangement
CHP	Cyclophosphamide, doxorubicin, and prednisone
CR	Complete remission
