# Supplementary material for: Impact of KMT2A Rearrangement on Peripheral T-Cell Lymphoma, Not Otherwise Specified, and Angioimmunoblastic T-Cell Lymphoma
Source: Biomedicines. 2025 Sep 25;13(10):2347. doi: 10.3390/biomedicines13102347 (PMC12562200; doi:10.3390/biomedicines13102347)
Supplement: Supplementary file 1 [file biomedicines-13-02347-s001.zip › biomedicines-3885146-supplementary.pdf]

Supplementary Materials

Table S1. Molecular alteration in the study cohort

Genes	Protein/Genomic change	Coding DNA change
DNMT3A	p.Arg882Cys, p.Arg688His, p.Gln415Ter	c.2644C>T, c.2063G>A, c.1243C>T
RHOA	p.Gly17Val	c.50G>T,
TET2	p.Cys1273Phe, p.Gln758Ter, p.Gln548Ter	c.2272C>T, c.1642C>T
ATM	p.Arg2993Ter	c.8977C>T
B2M	p.Met1?	c.1A>G
ARID1A	p.Gln581Ter	c.1741C>T
STAT6	p.Asn421Ser, p.Asn417Tyr, p.Asp419Gly	c.1262A>G, c.1249A>T, c.1256A>G
ATR	p.I774fs*5 g.142555898delT	c.2320delA
KRAS	p.Q61H g.25227341T>G, p.G13D, p.G12D	c.183A>C, c.38G>A, c.35G>A
TCF7L2	p.K485Sfs*23	c.1454delA
ERBB2	p.R217C g.39710091C>T	c.649C>T
IDH2	p.R140W, p.Arg42Gly, p.Arg172Lys, p.Arg172Met	c.418C>T, c.124A>G, c.515G>A, c.515G>T
BLM	p.N515Mfs*16	c.1544del
BRCA1	p.L52F	c.154C>T

VHL		c.341-2A>T
PIK3R1	p.R461_Y463delinsL	c.1382_1388delGAGAATAinsT
TP53	p.R248Q, p.Y220C, p.P152Afs*14, p.Gly127Ser, p.P151S	c.743G>A, c.659A>G, c.454_466del, c.379G>A, c.451C>T
SDHA	p.A449V	c.1346C>T
MLH1	p.R217C	c.649C>T
BRIP1	p.R814C	c.2440C>T, c.206-2A>T
KIT	p.T304A	c.910A>G
KMT2D	p.Asp4861Asn, p.P2662fs*29	c.14581G>A, c.7985delC, c.10441-6_10447delTTGCAGGAGCGGA
EZH2	p.Tyr646Phe	c.1937A>T
ARID1B	p.E1981fs*8	c.5942_5943delAG
**Fusion/Exon variants**		
FLT3	e19 : e21 fusion 15, e19 : e21 fusion 31, e9 : e11 fusion 32	
RET	e3 : e7 fusion 44, e6 : e8 fusion 55	
KMT2A	e5 : e7 fusion 14,, e5 : e7 fusion 38, e7 : e9 fusion 12, e7 : e9 fusion 24,e7 : e9 fusion 25 , e7 : e9 fusion 33, e7 : e9 fusion 40 ,e7 : e9 fusion 60, e7 : e9 fusion 68, e15 : e17 fusion 12, e15 : e17 fusion 13, e27 : e29 fusion 15,	
FGFR1	e3 : e4, e5 : e7, e7 : e9 fusion 91, 5'UTR	

ROS1	e32 : e34 fusion 133, e18 : e22 fusion 91, e8 : e10 fusion 235, e7 : e13 fusion 103, e7 : e10 fusion 151
**Copy number alteration**	
CDKN2A	Deletion 9p21.3
TNFAIP3	Deletion 6q23.3

Table S2. Univariate analysis of the overall survival outcomes of patients with PTCL-NOS and AITL

**Variables**	**HR (95% CI)**	**P-value**	**BH-FDR q-value**
PTCL-NOS vs. AITL	3.11 (1.00, 9.68)	0.051	0.229
Female vs. Male	0.43 (0.16, 1.17)	0.099	0.309
Age, > 60 years vs. ≤60 years	2.15 (0.81, 5.67)	0.123	0.309
Ann Arbor stage III–IV vs. I–II	1.44 (0.19, 11.0)	0.726	0.764
ECOG PS ≥ 2 vs <2	7.46 (1.94, 28.7)	0.003	0.069
LDH elevation vs normal	3.92 (1.12, 13.7)	0.033	0.217
Extranodal site involvement, ≥ 2 vs <2	1.48 (0.55, 4.01)	0.441	0.519
Bone marrow involvement, positive vs negative	2.58 (0.97, 6.87)	0.057	0.229
IPI score ≥3 vs <3	2.09 (0.79, 5.52)	0.139	0.309
TFH phenotype, yes or no	2.25 (0.63, 8.01)	0.209	0.373
Frontline regimen: BV-CHP vs. others	0.23 (0.03, 1.77)	0.159	0.319
EBER positive vs. negative	0.63 (0.24, 1.67)	0.354	0.476
ATR mutation vs. unmutated	1.04 (0.38, 2.85)	0.938	0.938
KMT2A rearranged, yes vs. no	2.54 (0.77, 8.42)	0.126	0.309
RHOA mutation vs. unmutated	1.6 (0.56, 4.55)	0.379	0.476
DNMT3A mutation vs. unmutated	3.45 (1.27, 9.36)	0.015	0.149

TCF7L2 mutation vs. unmutated	0.41 (0.05, 3.06)	0.381	0.476
IDH2 mutation vs. unmutated	2.01 (0.65, 6.17)	0.224	0.373
TP53 mutation vs. unmutated	0.4 (0.05, 3.01)	0.372	0.476
TET2 mutation vs. unmutated	1.59 (0.45, 5.57)	0.471	0.524

ECOG PS, Eastern Cooperative Oncology Group performance status; IPI, International Prognostic Index; EBER, EBV-encoded RNA in situ hybridization; BV-CHP, brentuximab vedotin + cyclophosphamide, doxorubicin, prednisone; AITL, angioimmunoblastic T-cell lymphoma; PTCL-NOS, peripheral T-cell lymphomas, not otherwise specified; TFH, T-follicular helper; LDH, Lactate dehydrogenase; HR, hazard ratio; CI, confidence interval; BH-FDR q-value, an adjusted p-value calculated using the Benjamini-Hochberg method to control the False Discovery Rate

Table S3. Multivariate analysis of the survival outcomes of patients with PTCL-NOS and AITL

Variables	HR (95% CI)	P-value
**Progression free survival**		
LDH elevation vs normal	2.32 (0.78, 6.94)	0.131
Bone marrow involvement, positive vs negative	1.45 (0.46, 4.55)	0.522
KMT2A rearranged, yes vs. no	1.65 (0.59, 4.59)	0.34
**Overall survival**		
ECOG PS $\geqslant$ 2 vs <2	5.24 (1.28, 21.5)	0.021
LDH elevation vs normal	2.60 (0.69, 9.76)	0.157
DNMT3A mutation vs. unmutated	2.62 (0.93, 7.36)	0.069

ECOG PS, Eastern Cooperative Oncology Group performance status; LDH, Lactate dehydrogenase; HR, hazard ratio; CI, confidence interval
